# Targeted proteomic and bioinformatic investigation of extracellular matrix remodeling in hAEC-EV-mediated corneal repair

**DOI:** 10.3389/fbioe.2026.1772203

**Published:** 2026-03-18

**Authors:** Shuqin Hu, Ting Qiu, Hong Liu

**Affiliations:** Department of Ophthalmology, Shanghai Children’s Medical Center, Shanghai Jiao Tong University School of Medicine, Shanghai, China

**Keywords:** corneal injury, extracellular matrix, extracellular matrix related genes, extracellular vesicles, human amniotic epithelial cell

## Abstract

**Introduction:**

Human amniotic epithelial cell-derived extracellular vesicles (hAEC-EVs) have shown therapeutic potential in corneal injury repair; however, the underlying molecular mechanisms, particularly those related to extracellular matrix (ECM) remodeling, remain incompletely understood.

**Methods:**

A proteomic and bioinformatic strategy was applied to analyze ECM-related molecular alterations in corneal tissues following alkali injury and hAEC-EV treatment. Differentially expressed ECM-related genes were identified and subjected to pathway enrichment, protein–protein interaction, and immune infiltration analyses. To enhance experimental rigor, key findings were validated at both the transcript and protein levels using quantitative real-time PCR and Western blotting. In addition, *in vitro* functional assays were performed to assess the effects of hAEC-EVs on corneal epithelial and stromal cell proliferation and migration.

**Results:**

hAEC-EV treatment significantly upregulated ECM-stabilizing molecules, including A2M, LAMA1, and VIT, while downregulating the injury- and inflammation-associated protease CTSB at both mRNA and protein levels. Enrichment analyses revealed that hAEC-EVs modulate ECM–receptor interaction pathways and cell–ECM communication. Functional assays confirmed that hAEC-EVs directly enhance the proliferation and migration of human corneal epithelial cells and human corneal stromal cells. Immune infiltration analysis further suggested that hAEC-EVs reshape the corneal immune microenvironment toward a repair-permissive state.

**Conclusion:**

Through integrated proteomic, bioinformatic, protein-level validation, and functional analyses, this study demonstrates that hAEC-EVs promote corneal repair by coordinating ECM remodeling, regulating key signaling networks, and modulating immune responses, providing mechanistic support for their therapeutic application in corneal injuries.

## Introduction

The cornea, located at the front of the eye, is a crucial transparent tissue that plays a vital role in refracting light for clear vision and acts as an important immune and mechanical barrier (Boote et al., 2003). Due to its exposure to the external environment, the cornea is vulnerable to a range of physical, mechanical, and chemical injuries. Presently, there is a lack of effective clinical interventions for treating corneal damage, particularly for alkali burns, which represent a prevalent and severely detrimental ophthalmic emergency. According to the World Health Organization (WHO), corneal blindness accounts for 5.1% of global blindness, ranking as the fourth leading cause after cataracts, glaucoma, and age-related macular degeneration. Hence, discovering efficacious treatments to facilitate corneal injury repair possesses immense clinical significance (Poon et al., 2020; Kumar et al., 2022).

In recent years, extracellular vesicles (EVs) have emerged as a promising biological therapeutic with potential in the repair of diverse tissue injuries (Herrera et al., 2010; Gatti et al., 2011; Zhang et al., 2015; Tao et al., 2017; Zhao et al., 2019). Released from nearly all prokaryotic and eukaryotic cells, EVs are membrane-enclosed nanoparticles that serve as vital components in paracrine cellular functions. These vesicles carry an array of bioactive substances, including proteins, lipids, and nucleic acids, contributing to intercellular signaling and tissue repair processes (Yáñez-Mó et al., 2015; Kalra et al., 2016). Our previous studies have demonstrated that hAEC-EVs can attenuate corneal inflammatory responses, facilitate re-epithelialization, orderly reconstruct the corneal stromal layer in rabbit corneal alkali burns, and reduce scar formation, thereby effectively promoting healing (Hu et al., 2022).

The ECM plays a pivotal role in corneal injury repair, not only providing structural support to cells but also influencing tissue regeneration and repair through modulation of cellular behavior and signal transduction. Post-injury, ECM remodeling and regeneration are essential steps in restoring corneal transparency and function. Research indicates that the composition and structural alterations of the ECM are closely linked to the corneal healing process, suggesting that regulating ECM synthesis and degradation could be an effective strategy to enhance corneal injury repair (Wilson et al., 2022).

In the present study, we employed proteomic and bioinformatic analyses to systematically investigate ECM-related molecular alterations during corneal injury repair mediated by hAEC-EVs. To address methodological limitations of previous omics-based studies, we complemented bioinformatic predictions with protein-level validation using Western blotting and transcript-level confirmation by qPCR. Furthermore, we performed *in vitro* functional assays to directly evaluate the effects of hAEC-EVs on corneal epithelial and stromal cell proliferation and migration. Through this integrated approach, we aimed to provide a more rigorous and comprehensive mechanistic framework for hAEC-EV–mediated corneal repair.

## Materials and methods

### Isolation and identification of hAEC-EVs

All hAEC-EV preparations used in this study were produced and quality-controlled using the same standardized protocol as previously reported (Hu et al., 2022). HAECs at passage 3 were expanded until reaching approximately 80%–90% confluence. Cells were rinsed three times with PBS and subsequently cultured in DMEM/F-12 medium supplemented with 5% EV-depleted FBS for 48 h. EV-depleted FBS was prepared by ultracentrifugation of FBS as previously reported. hAEC-EVs were isolated from conditioned medium by differential ultracentrifugation. Briefly, supernatants were sequentially centrifuged at 300 × g for 10 min, 2000 × g for 20 min, and 10,000 × g for 30 min at 4 °C, followed by filtration through a 0.22-μm membrane. The filtrate was ultracentrifuged at 150,000 × g for 2 h at 4 °C using an SW70Ti rotor (Beckman Coulter, USA). The hAEC-EV pellet was washed once with PBS under the same conditions and finally resuspended in 200 μL PBS.

Protein concentration was measured using a BCA assay (BCA, 23227, Thermo Fisher Scientific, USA). The hAEC-EV particle sizes were determined using nanoparticle tracking analysis (NTA) with ZetaView PMX 110 (Particle Metrix, Meerbusch, Germany) and ZetaView 8.04.02 software. According to the NTA and BCA assays, the concentration of hAEC-EVs was 1.5 × 107 particles/μg. EV morphology and marker expression were characterized by transmission electron microscopy (TEM, HT-7700, Hitachi, Japan) and Western blotting. These results have been fully documented in our previous publication and are therefore not presented again in the present manuscript (Hu et al., 2022).

### Animal experiments

All *in vivo* procedures complied with the Guide for the Care and Use of Laboratory Animals. Ethical approval for animal use was granted by the Institute of Laboratory Animal Resources of Tongji University (Approval No. TJAA09620602), and all experiments conformed to the ARVO guidelines for animal research in ophthalmology and vision science. Male rabbits weighing between 2.0 and 2.5 kg were selected for this study. At the conclusion of the experiments, animals were humanely sacrificed by administering an excessive dose of pentobarbital intravenously.

To establish the corneal alkali injury model, a sterile ring-shaped Whatman filter paper (6 mm in diameter; GE Healthcare Life Sciences, USA) soaked with 10 μL of 1 N NaOH was applied to the central cornea for 20 s. Immediately afterward, the injured cornea was thoroughly irrigated with 40 mL of sterile saline. Rabbits with successful corneal alkali injuries were randomly assigned to either the PBS control group or the hAEC-EV treatment group (n > 6). Animals in the hAEC-EV group received topical hAEC-EVs (1 mg/mL, 40 μL) three times per day along with subconjunctival injections of hAEC-EVs (1 mg/mL, 100 μL) administered twice weekly, whereas control animals were treated with an equivalent volume of 1× PBS. The total duration of the treatment regimen was 2 weeks. This treatment regimen was selected based on our previously optimized protocol (Hu et al., 2022).

### Cell culture

To obtain and maintain hAECs, the amniotic membrane was first manually separated from the chorion and rinsed three times with PBS (E607008, Sangon Biotech, China) containing 10% penicillin–streptomycin (15070063, Thermo Fisher Scientific, USA). The isolated amnion was then digested with 0.25% trypsin–EDTA (25200072, Thermo Fisher Scientific, USA) at 37 °C for 15 min, after which epithelial cells were gently released by mechanical agitation. The resulting cell suspension was harvested by centrifugation at 1,500 rpm for 10 min and seeded into 10-cm culture dishes containing DMEM/F-12 medium (11330032, Thermo Fisher Scientific, USA) supplemented with 10% FBS (ExCell Bio, China), 10 ng/mL recombinant human epidermal growth factor (EGF; PHG0311, Thermo Fisher Scientific, USA), 5 μM SB431542 (301836-41-9, Selleck, China), and 1% penicillin–streptomycin. Cells were maintained at 37 °C in a humidified incubator, and the culture medium was refreshed every other day.

HCECs and HCSCs were generously provided by Professor Qingjun Zhou (Shandong Eye Institute, China). These cells were expanded in DMEM/F-12 medium supplemented with 10% FBS and cultured under standard conditions at 37 °C.

### Data download

We conducted the analysis using the self-assessed datasets, Dataset-NP and Dataset-PH, with all samples derived from New Zealand White Rabbits (*Oryctolagus cuniculus*). Dataset-NP comprises three samples originating from the healthy group (Normal) and three samples originating from the PBS group (PBS). Meanwhile, Dataset-PH consists of three samples originating from the PBS group (PBS) and three samples originating from the hAEC-EVs group (hAEC-EVs). All samples from both Dataset-NP and Dataset-PH were included in this study. Detailed information is provided in Table 1.

**TABLE 1:** Dataset information list.

Characteristics	Dataset-NP	Dataset-PH
Species	*Oryctolagus cuniculus*	*Oryctolagus cuniculus*
Samples in test group	PBS:3	hAEC-EVs:3
Samples in control group	Normal:3	PBS:3

Extracellular Matrix Related Genes (ECMRGs) were gathered from the GeneCards database (Stelzer et al., 2016) (https://www.genecards.org/), which offers detailed information on human genes. Using “Extracellular Matrix” as the search term, after only keeping “Protein Coding” and “Relevance Score >5” ECMRGs, 501 ECMRGs were obtained.

We first obtained the expression profile matrix of the corresponding human genes through the self-test Dataset, and obtained the self-test datasets Dataset-NP and Dataset-PH. Subsequently, the R package limma (Ritchie et al., 2015) was employed to normalize the self-test datasets, Dataset-NP and Dataset-PH, with the normalized data then utilized for further analysis.

### Corneal injury-related extracellular matrix related differentially expressed genes (ECMRDEGs)

According to the sample classification in the self-test datasets, Dataset-NP and Dataset-PH, samples were categorized into the Normal group and PBS group, and into the PBS group and hAEC-EVs group, respectively. The R package limma was employed to examine the gene expression differences between the Normal group and the PBS group, as well as between the PBS group and the hAEC-EVs group. To identify Differentially Expressed Genes (DEGs), a threshold of |logFC| > 0 and p.value <0.05 was set. Genes showing logFC >0 and p.value <0.05 were labeled as Upregulated, whereas genes with logFC <0 and p.value <0.05 were marked as Downregulated. The results of the differential analysis were illustrated using volcano plots generated with the R package ggplot2.

To identify ECMRDEGs related to corneal injury, we combined all DEGs with |logFC| > 0 and p.value <0.05 derived from analyzing Dataset-NP and Dataset-PH with ECMRGs. A Venn diagram was then plotted to visualize the ECMRDEGs.

### Gene set enrichment analysis (GSEA)

GSEA (Subramanian et al., 2005) assesses the distribution pattern of predefined gene sets within a gene list that is ranked based on their correlation with a phenotype, thus evaluating their impact on the phenotype. In this research, genes from Dataset-NP and Dataset-PH were ranked according to logFC values. Using the R package clusterProfiler, GSEA was performed on all genes in both datasets. The parameters for GSEA were set as follows: seed is 2020, the number of computations is 1,000, the minimum number of genes contained in each gene set is 10, and the maximum number of genes contained in each gene set is 500. For the analysis, the gene set c2.cgp.v7.5.1.symbols.gmt [Chemical and Genetic Perturbations] (3,384) was sourced from the from the Molecular Signatures Database (MSigDB) 3.0 (Liberzon et al., 2011). The criteria used to select for GSEA included p.adj <0.05 and FDR (q value) <0.05, with p-value adjustments made using the Benjamini-Hochberg (BH) method.

### Gene set variation analysis (GSVA)

GSVA (Hanzelmann et al., 2013) is an unsupervised, non-parametric approach that converts the gene expression matrix from various samples into a matrix reflecting gene set expressions, allowing for the assessment of gene set enrichment in microarray transcriptomes. It determines if different pathways are enriched in the samples. The gene set c8.all.v2023.2.Hs.symbols.gmt was obtained from the MSigDB for GSVA analysis on all genes in Dataset-NP and Dataset-PH. The study evaluated functional enrichment differences between groups, applying a selection criterion of p.adj <0.05 and adjusting p-values using the Benjamini-Hochberg (BH) method.

### Gene ontology (GO) and pathway (KEGG) enrichment analysis

GO (Mi et al., 2019) analysis is extensively used for comprehensive functional enrichment studies, including Biological Process (BP), Cellular Component (CC), and Molecular Function (MF). The Kyoto Encyclopedia of Genes and Genomes (KEGG) (Kanehisa and Goto, 2000) is a detailed database that contains information on genomes, biological pathways, diseases, and drugs. We utilized the R package clusterProfiler (Yu et al., 2012) to conduct GO and KEGG enrichment analyses on ECMRDEGs. Entries with p.adj <0.05 and FDR (q value) <0.05 were considered statistically significant, with p-values adjusted using the BH method.

### Protein-protein interaction (PPI) network

The PPI network is a complex web created by interactions between proteins, which are essential for biological signal transduction, regulation of gene expression, metabolism of energy and substances, and cell cycle management. Analyzing these interactions systematically is essential for understanding protein function mechanisms, physiological response mechanisms in conditions like diseases, and the relationships between proteins. The STRING database (Szklarczyk et al., 2019) (https://cn.string-db.org/) serves as a resource for both known and predicted protein interactions. In our study, we utilized the STRING database to build a PPI network based on ECMRDEGs, setting the minimum interaction score at 0.400 for medium confidence. Clusters within this network could indicate molecular complexes with distinct biological functions. We identified genes that interact with others in the network as hub genes for further analysis. We used Cytoscape software (Shannon et al., 2003) to create a visual representation of the network.

### Construct mRNA-miRNA and mRNA-TF interaction networks

MicroRNAs are essential in regulating biological evolution, as they can influence numerous target genes, and a single gene may be controlled by several microRNAs. o examine the connection between Hub Genes and miRNAs, we retrieved relevant miRNAs from the miRDB database (Chen and Wang, 2020) and, after filtering, used Cytoscape software to visualize the mRNA-miRNA regulatory network.

Transcription Factors (TFs) regulate gene expression post-transcriptionally through interactions with Hub Genes. We obtained transcription factors from the ChIPBase database (Zhou et al., 2017) to study their regulatory impact on Hub Genes and, following filtering, used Cytoscape software to visualize the mRNA-TF regulatory network.

### Immunoinfiltration analysis

Single-Sample Gene-Set Enrichment Analysis (ssGSEA) (Xiao et al., 2020) measures the relative levels of immune cell infiltration. Initially, different types of infiltrating immune cells are identified, including Activated dendritic cells, Natural killer cells, Activated CD8 T cells, Gamma delta T cells, and Regulatory T cells, along with other human immune subtypes. Subsequently, ssGSEA computes enrichment scores to indicate the relative levels of immune cell infiltration in each sample, resulting in an immune cell infiltration matrix. Immune cells with significant differences between groups are selected for further analysis. The correlation among immune cells is computed using the Spearman algorithm, and the correlation heatmap is generated with the R package pheatmap to display results. The relationship between ECMRDEGs and immune cells is also calculated using the Spearman algorithm, retaining results with a p.value <0.05, and a correlation bubble chart is created with the R package ggplot2 to illustrate the analysis results.

### qPCR validation of ECM-related genes

Total RNA was isolated from corneal tissues of the Normal, PBS, and hAEC-EVs treatment groups. Reverse transcription was performed using the PrimeScript™ RT Master Mix kit (Takara, Shiga, Japan) according to the manufacturer’s instructions. Quantitative real-time PCR was conducted on a Chromo4 instrument (Bio-Rad, Hercules, USA) utilizing the SuperReal Premix Plus kit (Tiangen Biotech, Beijing, China) with SYBR Green detection. Amplification conditions consisted of an initial denaturation at 95 °C for 5 min, followed by 40 cycles of 95 °C for 30 s and 60 °C for 30 s. Specific primers for target genes A2M, LAMA1, VIT, COL15A1, TNS1, TGM2, CTSB, ITGB6, TIMP1, and VCAN (synthesized by Sangon Biotech Co., Ltd., Shanghai, China) were listed in Table 2, with GAPDH serving as the internal control. All reactions were run in triplicate to ensure reproducibility.

**TABLE 2:** Primers and their sequences for qRT-PCR analysis.

Gene symbol	Forward (5′-3′)	Reverse (5′-3′)
*A2M*	GGT​GGT​GGA​GAA​GGA​TTT​ATT	TTG​GAC​AGT​GAG​GAA​CAT​TAC
*LAMA1*	AGA​CAG​GAG​CAA​GAA​GTA​GA	TGT​TGC​TAA​GGA​CAG​ACA​TAA​A
*VIT*	CAC​TGA​TGG​GTG​TTG​TTC​AG	CCC​TCC​TCT​CTG​GGT​TAT​TT
*COL15A1*	CCT​GTT​CTG​AGT​GCC​AAT​TA	CTGCTGGAAGCACTGAAA
*TNS1*	GAG​GAC​TTT​GGG​CTG​ATT​T	TTC​CTC​TCT​CTC​TCT​CTC​TTT​C
*TGM2*	CTG​ACA​CAG​TCC​AAC​CTT​ATC	GGA​TCT​TGA​TTT​CGG​GAT​TCT
*CTSB*	CGG​CAT​GGT​TCC​TTT​CTA​ATA	GAC​AAT​CTG​GAA​AGG​GAG​TG
*ITGB6*	CAC​TAT​GCC​TGT​GGA​AGA​AA	TTA​TCC​TTG​CGG​TGG​TAA​TG
*TIMP1*	CGA​AGC​CTA​CAC​CAT​GTT​T	GCA​GGG​TGG​TAG​TTC​TTT​ATT
*VCAN*	GGA​ACC​GTA​GAG​GTC​TTT​AAT​C	TCCAAGGGCAAAGGAAAC

### Western blotting analysis

Corneal tissues cells were lysed using RIPA buffer (P0013B, Beyotime, China) with the addition of protease and phosphatase inhibitors (C0001 and C0004, TargetMol, USA). Twenty micrograms of total protein denatured in loading buffer (Yeasen, China) were separated using SDS-PAGE and transferred to a 0.45 μm PVDF membrane (Merck, Germany). The membranes were blocked in 5% BSA for 1 h at RT, incubated with primary antibodies as shown in Table 3 at 4 °C overnight and incubated with horseradish peroxidase (HRP)-conjugated secondary antibodies for 2 h at RT. Protein bands were examined using the PierceTM ECL Western blotting substrate (Thermo Fisher Scientific, USA) and a Tanon chemiluminescence image detection system (5200S, Tanon, China).

**TABLE 3:** Antibodies used in WB.

Antibodies	Cat no.	Brand	Dilution ratio
A2M	13545-1-AP	Proteintech	1:1,000
LAMA1	28697-1-AP	Proteintech	1:1,000
VIT	abx025749	Abbexa	1:1,000
COL15A1	HW534014	Abinscience	1:1,000
TNS1	20054-1-AP	Proteintech	1:1,000
TGM2	15100-1-AP	Proteintech	1:1,000
CTSB	A19005	Abclonal	1:1,000
TIMP1	16644-1-AP	Proteintech	1:1,000
VCAN	30599-1-AP	Proteintech	1:1,000

### Cell proliferation assay

Cultured HCECs or HCSCs were plated into 48-well plates and allowed to adhere for 24 h. Cells were then treated with serum-free DMEM/F-12 alone as the control or with the same medium containing hAEC-EVs at a final concentration of 100 μg/mL (total protein). Following an additional 24 h of incubation, cell proliferation was assessed using a 5-ethynyl-2′-deoxyuridine (EdU) incorporation assay in accordance with the manufacturer’s protocol (EdU Cell Proliferation Kit, C0071S, Beyotime, China). Fluorescently labeled cells were observed and imaged using a fluorescence microscope (Olympus, Japan).

### Transwell migration assay

Vertical migration of HCSCs or HCECs was evaluated using polycarbonate Transwell inserts with an 8-µm pore size. For each assay, 500 µL of serum-free DMEM/F-12 was placed in the lower chamber of a 24-well plate as the control, or the same medium supplemented with hAEC-EVs at a final protein concentration of 100 μg/mL. HCECs or HCSCs were suspended in serum-free culture medium, and 200 µL of the cell suspension containing 5 × 10^4^ cells was seeded onto the upper surface of the Transwell membrane. After 24 h of incubation, migrated cells were fixed and stained with 0.5% crystal violet (60506ES60, Yeasen, China). Cells that traversed to the underside of the insert were observed and imaged using a light microscope (Olympus, Japan). Migratory cells were quantified by counting three nonoverlapping microscopic fields per insert, and the results were subjected to statistical analysis.

### Statistical analysis

For all quantitative data, the mean ± SEM was expressed. The data were evaluated and presented with GraphPad Prism 9.5 (GraphPad Software, USA). The statistical comparison was evaluated with one-way ANOVA. P < 0.05 was deemed statistically significant. In the graphs, asterisks are displayed to indicate the statistical significance of the values. *: P < 0.05; **: P < 0.01; ***: P < 0.001; ****: P < 0.0001.

## Results

### Differential expression analysis of corneal injury-related extracellular matrix

**FIGURE 1:**
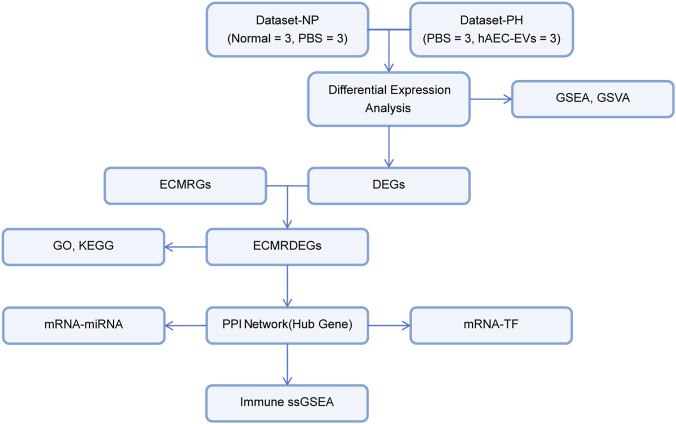
Technology Roadmap. Abbreviations: DEGs: Differentially Expressed Genes; ECMRGs: Extracellular Matrix Related Genes; ECMRDEGs: Extracellular Matrix Related Differentially Expressed Genes; GSEA: Gene Set Enrichment Analysis; GSVA: Gene Set Variation Analysis; PPI Network: Protein-Protein Interaction Network; TF: Transcription Factors; GO: Gene Ontology; KEGG: Kyoto Encyclopedia of Genes and Genomes; ssGSEA: Single-sample Gene Set Enrichment Analysis.

**FIGURE 2:**
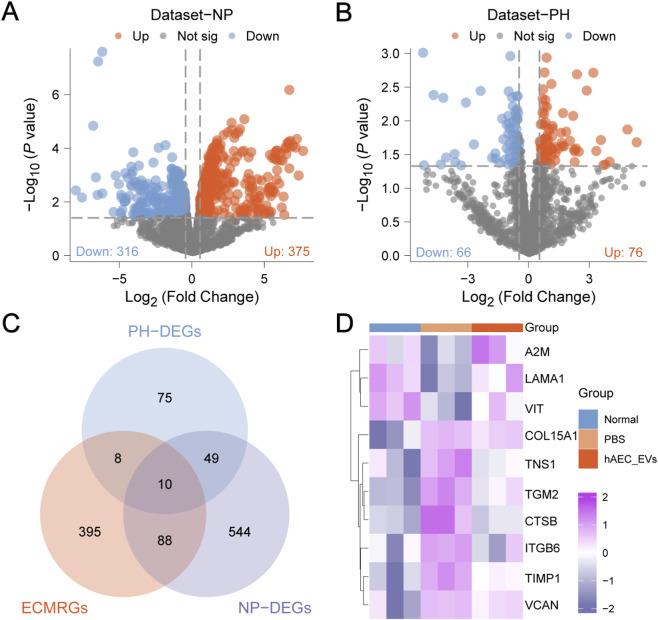
Differential expression analysis of Dataset-NP and Dataset-PH. **(A)** Volcano plot of differential gene analysis between PBS and Normal samples in Dataset-NP. **(B)** Volcano plot of differential gene analysis between PBS and hAEC-EVs samples in Dataset-PH. **(C)** Venn diagram of DEGs and ECMRGs in Dataset-NP and Dataset-PH. **(D)** Heatmap of ECMRDEGs. Orange represents the PBS group, blue for the Normal group, and purple for the hAEC-EVs group. In the heatmap, purple indicates high expression, while dark purple signifies low expression.

The overall experimental framework and technical roadmap of this study are illustrated in Figure 1. To examine the differences in gene expression values between groups in the self-assessed datasets Dataset-NP and Dataset-PH, the R package limma was used for differential analysis. The findings are as follows: Dataset-NP identified 691 differentially expressed genes (DEGs) that met the criteria of |logFC| > 0 and p.value <0.05. Among these, 375 genes were upregulated (logFC >0 and p.value <0.05), while 316 were downregulated (logFC <0 and p.value <0.05). A volcano plot illustrating these results was generated (Figure 2A). In Dataset-PH, 142 DEGs met the threshold of |logFC| > 0 and p.value <0.05, with 76 upregulated (logFC >0 and p.value <0.05) and 66 downregulated (logFC <0 and p.value <0.05). A corresponding volcano plot was also produced for this dataset (Figure 2B). To identify DEGs related to the ECMRDEGs linked to corneal injury, we intersected all DEGs with |logFC| >0 and p.value <0.05 from both Dataset-NP and Dataset-PH with ECMRGs. A Venn diagram was created to depict this intersection (Figure 2C). This analysis resulted in 10 ECMRDEGs: TIMP1, TNS1, TGM2, CTSB, VCAN, A2M, LAMA1, VIT, ITGB6, and COL15A1. A heatmap was then produced using the R package pheatmap to present these results (Figure 2D).

### qPCR and western blotting validation of key ECM-related genes

**FIGURE 3:**
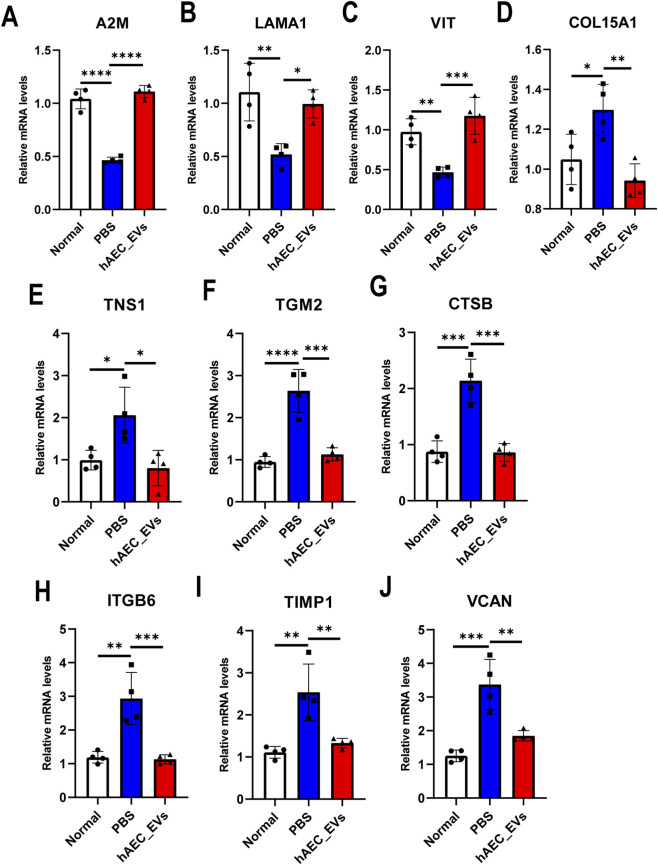
qPCR validation of ECM-related gene expression. **(A–J)** The mRNA expression levels of A2M, LAMA1, VIT, COL15A1, TNS1, TGM2, CTSB, ITGB6, TIMP1, and VCAN were quantified in samples from the Normal, PBS, and hAEC-EVs groups using qRT-PCR. Statistical analysis was performed using one-way ANOVA. Data are presented as the means ± SEM (n = 3). *: P < 0.05; **: P < 0.01; ***: P < 0.001; ****: P < 0.0001.

To experimentally verify the ECMRDEGs identified in the proteomic and bioinformatic analyses, both qPCR and Western blotting were performed on selected key genes. qPCR results demonstrated that the expression of A2M, LAMA1, and VIT was significantly increased in the hAEC-EVs group compared with the PBS group. Among these, A2M and LAMA1 exhibited the most pronounced elevation, consistent with their central roles in maintaining ECM structural homeostasis. Conversely, CTSB, a gene closely associated with inflammation and tissue injury, was markedly downregulated following hAEC-EVs treatment (Figures 3A–J).

**FIGURE 4:**
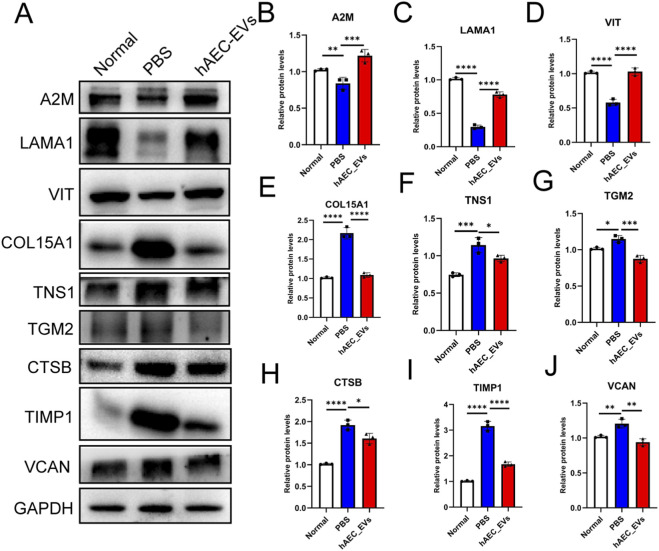
Western blot validation of ECM-related gene expression at the protein level. **(A–J)** The protein expression levels of A2M, LAMA1, VIT, COL15A1, TNS1, TGM2, CTSB, TIMP1, and VCAN were analyzed in samples from the Normal, PBS, and hAEC-EVs groups by Western blot. Protein band intensities were quantified and normalized to GAPDH. Statistical analysis was performed using one-way ANOVA. Data are presented as the means ± SEM (n = 3). *: P < 0.05; **: P < 0.01; ***: P < 0.001; ****: P < 0.0001.

Importantly, Western blotting analysis further confirmed these findings at the protein level. Consistent with the qPCR and proteomic data, the protein expression levels of A2M, LAMA1, and VIT were significantly upregulated in the hAEC-EVs–treated corneas, whereas CTSB protein expression was substantially reduced compared with the PBS group (Figures 4A–J). Quantitative densitometric analysis revealed expression patterns that closely mirrored the transcriptomic and proteomic trends, demonstrating strong concordance between mRNA and protein levels.

Together, the combined qPCR and Western blotting results provide robust molecular evidence supporting the regulatory effects of hAEC-EVs on ECM remodeling. These protein-level validations effectively bridge the gap between proteomic screening and functional interpretation, thereby strengthening the scientific rigor and reliability of the conclusions drawn from the proteomic analyses.

### GSEA

**FIGURE 5:**
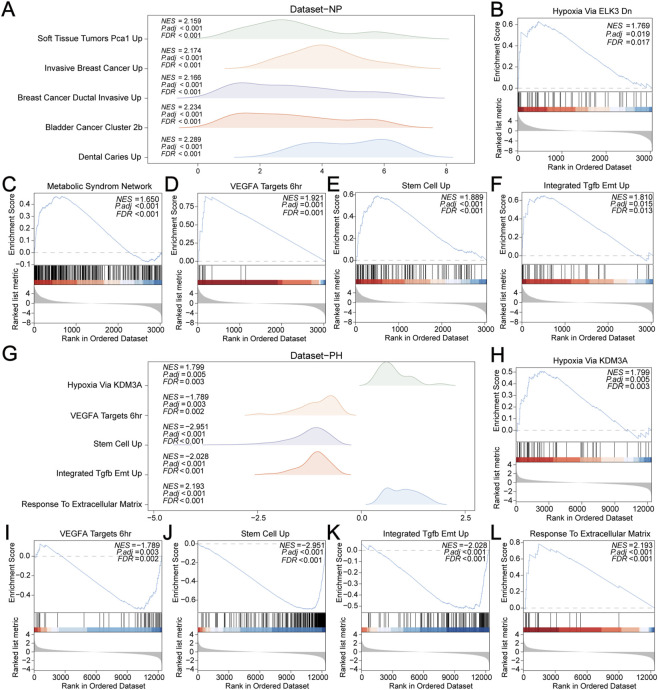
GSEA Analysis of Dataset-NP and Dataset-PH. **(A)** GSEA ridge plots of five biological functions in Dataset-NP. **(B–F)** GSEA reveals significant enrichment of all genes in HYPOXIA_VIA_ELK3_DN **(B)**, METABOLIC_SYNDROME_NETWORK **(C)**, VEGFA_TARGETS_6HR **(D)**, STEM_CELL_UP **(E)**, INTEGRATED_TGFB_EMT_UP **(F)**. **(G)** GSEA ridge plots of five biological functions in Dataset-PH. (H–L) GSEA shows significant enrichment of all genes in HYPOXIA_VIA_KDM3A **(H)**, VEGFA_TARGETS_6HR **(I)**, STEM_CELL_UP **(J)**, INTEGRATED_TGFB_EMT_UP **(K)**, RESPONSE_TO_EXTRACELLULAR_MATRIX **(L)**. GSEA selection criteria were p.adj <0.05 and FDR (q value) < 0.05, with p.value adjusted by the Benjamini-Hochberg (BH) method.

GSEA was performed to determine the effect of gene expression levels on corneal injury within the self-assessed datasets, Dataset-NP and Dataset-PH. This analysis examined the relationship between gene expression and their roles in biological processes, cellular components, and molecular functions in Dataset-NP (Figure 5A) and Dataset-PH (Figure 5G). The analysis indicated that genes in Dataset-NP were significantly enriched in biological functions and signaling pathways such as HYPOXIA_VIA_ELK3_DN (Figure 5B), METABOLIC_SYNDROME_NETWORK (Figure 5C), VEGFA_TARGETS_6HR (Figure 5D), STEM_CELL_UP (Figure 5E), and INTEGRATED_TGFB_EMT_UP (Figure 5F). Likewise, genes in Dataset-PH were significantly enriched in pathways including HYPOXIA_VIA_KDM3A (Figure 5H), VEGFA_TARGETS_6HR (Figure 5I), STEM_CELL_UP (Figure 5J), INTEGRATED_TGFB_EMT_UP (Figure 5K), and RESPONSE_TO_EXTRACELLULAR_MATRIX (Figure 5L).

### GSVA

**FIGURE 6:**
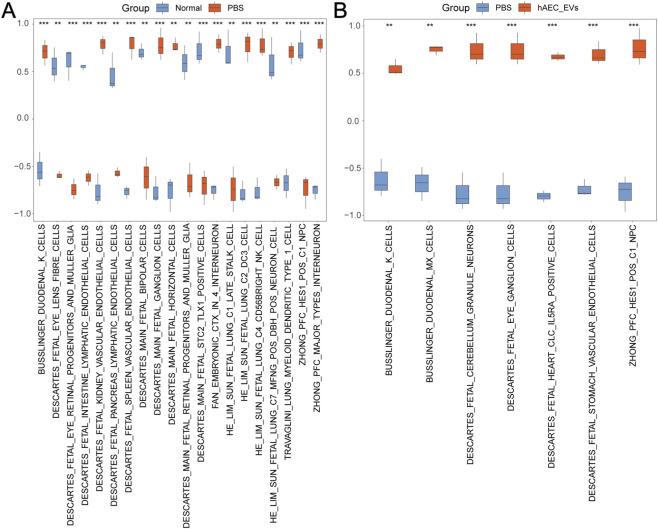
GSVA Analysis of Dataset-NP and Dataset-PH. **(A)** Group comparison plot of GSVA results between the Normal and PBS groups in Dataset-NP. **(B)** Group comparison plot of GSVA results between the PBS and hAEC-EVs groups in Dataset-PH. **: P < 0.01; ***: P < 0.001. GSVA selection criteria were p.adj <0.05, with p.value adjusted by the Benjamini-Hochberg (BH) method. In Dataset-NP, orange represents the PBS group, blue the Normal group. In Dataset-PH, orange denotes the hAEC-EVs group, blue the PBS group.

GSVA was conducted on all genes in both datasets to investigate the differences in the c8.all.v2023.2.Hs.symbols.gmt gene set between the Normal and PBS groups in Dataset-NP, as well as between the hAEC-EVs and PBS groups in Dataset-PH. In Dataset-NP, the top 10 positively enriched pathways and the top 10 negatively enriched pathways, ranked by logFC with p.adj <0.05, were selected. The differential expression of these 20 pathways between the Normal and PBS groups was confirmed using a T-test, and the results were depicted in a comparison plot (Figure 6A). The results of the GSVA revealed that the following pathways exhibited statistical significance (p.value <0.05) between the Normal and PBS groups: ZHONG_PFC_HES1_POS_C1_NPC, HE_LIM_SUN_FETAL_LUNG_C1_LATE_STALK_CELL, DESCARTES_MAIN_FETAL_STC2_TLX1_POSITIVE_CELLS, DESCARTES_FETAL_EYE_RETINAL_PROGENITORS_AND_MULLER_GLIA, DESCARTES_MAIN_FETAL_BIPOLAR_CELLS, HE_LIM_SUN_FETAL_LUNG_C7_MFNG_POS_DBH_POS_NEURON_CELL, DESCARTES_MAIN_FETAL_RETINAL_PROGENITORS_AND_MULLER_GLIA, DESCARTES_FETAL_INTESTINE_LYMPHATIC_ENDOTHELIAL_CELLS, DESCARTES_FETAL_EYE_LENS_FIBRE_CELLS, DESCARTES_FETAL_PANCREAS_LYMPHATIC_ENDOTHELIAL_CELLS, BUSSLINGER_DUODENAL_K_CELLS, TRAVAGLINI_LUNG_MYELOID_DENDRITIC_TYPE_1_CELL, DESCARTES_MAIN_FETAL_HORIZONTAL_CELLS, DESCARTES_MAIN_FETAL_GANGLION_CELLS, HE_LIM_SUN_FETAL_LUNG_C4_CD56BRIGHT_NK_CELL, FAN_EMBRYONIC_CTX_IN_4_INTERNEURON, ZHONG_PFC_MAJOR_TYPES_INTERNEURON, DESCARTES_FETAL_KIDNEY_VASCULAR_ENDOTHELIAL_CELLS, DESCARTES_FETAL_SPLEEN_VASCULAR_ENDOTHELIAL_CELLS, and HE_LIM_SUN_FETAL_LUNG_C2_DC3_CELL.

In Dataset-PH, pathways with p.adj <0.05 were selected. A comparison plot was created (Figure 6B) to illustrate the results. The Gene Set Variation Analysis (GSVA) indicated that the pathways ZHONG_PFC_HES1_POS_C1_NPC, DESCARTES_FETAL_CEREBELLUM_GRANULE_NEURONS, DESCARTES_FETAL_EYE_GANGLION_CELLS, DESCARTES_FETAL_HEART_CLC_IL5RA_POSITIVE_CELLS, DESCARTES_FETAL_STOMACH_VASCULAR_ENDOTHELIAL_CELLS, BUSSLINGER_DUODENAL_MX_CELLS, and BUSSLINGER_DUODENAL_K_CELLS showed statistical significance (p.value <0.05) between the PBS and hAEC-EVs groups.

Additionally, the pathways BUSSLINGER_DUODENAL_K_CELLS and ZHONG_PFC_HES1_POS_C1_NPC were statistically significant (p.value <0.05) across both datasets. Notably, the pathway ZHONG_PFC_HES1_POS_C1_NPC was highly expressed in the Normal and hAEC-EVs groups, and lowly expressed in the PBS group. It should be noted that some GSVA-enriched signatures may reflect context-dependent transcriptional similarity rather than tissue-specific cell identities and therefore should be interpreted with caution.

### GO and KEGG enrichment analysis

**FIGURE 7:**
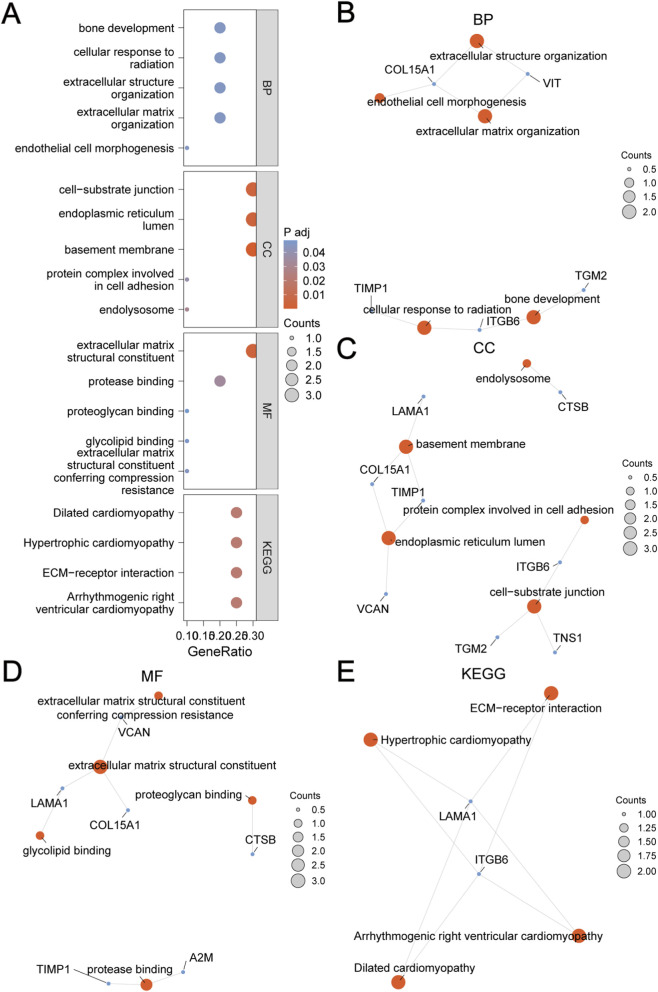
GO and KEGG Enrichment Analysis. **(A)** Bubble plot displaying the enrichment analysis results of ECMRDEGs for GO and KEGG pathway: BP, CC, MF, and biological pathways (KEGG). The vertical axis represents GO and KEGG terms. **(B–E)** Network diagrams of ECMRDEGs’ GO enrichment analysis: BP **(B)**, CC **(C)**, MF **(D)**, and KEGG **(E)**. Red nodes represent terms, blue nodes signify molecules, and lines denote the relationship between terms and molecules. Abbreviations: BP: Biological Process; CC: Cellular Component; MF: Molecular Function; In the bubble plot, the bubble size indicates the number of genes, and the bubble color reflects the p.adj value, with redder hues indicating smaller p.adj values and bluer hues indicating larger p.adj values. The selection criteria for GO and KEGG enrichment analysis were p.adj <0.05 and FDR (q value) < 0.05, with p.value adjusted using the BH method.

To explore the connection between ECMRDEGs and corneal injury in Dataset-NP and Dataset-PH, we performed enrichment analyses using GO and KEGG pathways. The analyses indicated that the 10 ECMRDEGs are mainly enriched in biological processes such as extracellular matrix organization, extracellular structure organization, cellular response to radiation, endothelial cell morphogenesis, and bone development. Regarding cellular components, they are linked to the basement membrane, endoplasmic reticulum lumen, cell-substrate junction, endolysosome, and protein complexes involved in cell adhesion. The molecular functions include extracellular matrix structural constituent, protease binding, extracellular matrix structural constituent conferring compression resistance, glycolipid binding, and proteoglycan binding. Furthermore, they are enriched in KEGG pathways like ECM-receptor interaction, Arrhythmogenic right ventricular cardiomyopathy, Hypertrophic cardiomyopathy, and Dilated cardiomyopathy. A bubble plot (Figure 7A) visualizes the results of the GO and KEGG enrichment analyses.

Additionally, network diagrams for biological processes (BP), cellular components (CC), and KEGG pathways have been created based on the enrichment analysis results (Figures 7B–E). The lines illustrate the annotations between related molecules and items, while larger nodes signify items that encompass a greater number of molecules.

### The PPI network, mRNA-miRNA and mRNA-TF interaction network were constructed

**FIGURE 8:**
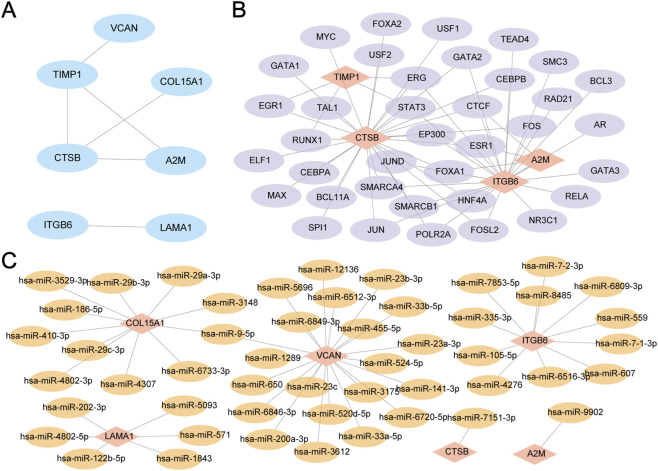
Construction of PPI Interaction Network. **(A)** PPI Network of ECMRDEGs computed using the STRING database. **(B)** mRNA-TF Regulatory Network for Hub Genes. **(C)** mRNA-miRNA Regulatory Network for Hub Genes. Abbreviations: PPI Network: Protein-Protein Interaction Network; TF: Transcription Factor. Red represents mRNA, purple indicates TF, and orange signifies miRNA.

To begin, perform a protein-protein interaction analysis utilizing the STRING database to develop a PPI Network for the 10 ECMRDEGs (Figure 8A). The results of the PPI Network reveal connections among seven ECMRDEGs, which are identified as Hub Genes: A2M, COL15A1, CTSB, ITGB6, LAMA1, TIMP1, and VCAN. Subsequently, extract transcription factors (TFs) linked to the Hub Genes from the ChIPBase database to create the mRNA-TF Regulatory Network, which is visualized using Cytoscape software (Figure 8B). This network comprises four Hub Genes and 37 TFs. Lastly, obtain miRNAs associated with the Hub Genes from the miRDB database to construct the mRNA-miRNA Regulatory Network, also visualized with Cytoscape software (Figure 8C). This network includes six Hub Genes and 50 miRNA.

### Immune infiltration analysis

**FIGURE 9:**
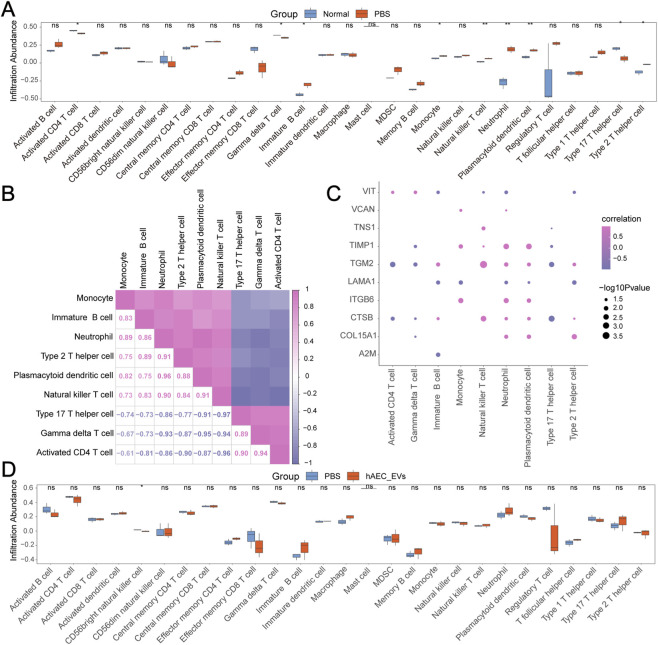
Immune Infiltration Analysis of Dataset-NP and Dataset-PH. **(A)** Comparative plot of immune cells in the Normal and PBS groups within Dataset-NP. **(B)** Correlation heatmap of immune cell infiltration abundance in Dataset-NP. **(C)** Correlation scatter plot of ECMRDEGs with immune cell infiltration abundance in Dataset-NP. **(D)** Comparative plot of immune cells in the PBS and hAEC-EVs groups within Dataset-PH. Abbreviations: ssGSEA: Single-sample Gene Set Enrichment Analysis. Correlation coefficient (r value) below 0.3 denotes weak or no correlation, between 0.3 and 0.5 indicates weak correlation, and between 0.5 and 0.8 suggests moderate correlation. Pink represents positive correlation, purple signifies negative correlation. The intensity of color indicates the strength of the correlation. In Dataset-NP, red denotes the PBS group, blue the Normal group. In Dataset-PH, red indicates the hAEC-EVs group, blue the PBS group.

Using Dataset-NP, the ssGSEA algorithm was employed to determine the infiltration abundance of 28 types of immune cells. Following the immune infiltration analysis, a T-test was performed for differential validation, and a comparative plot was generated to illustrate expression differences in immune cell infiltration across various groups (Figure 9A). The findings indicated significant statistical differences (p.value <0.05) in nine immune cells among different groups in Dataset-NP, including Activated CD4 T cells, Gamma delta T cells, Immature B cells, Monocytes, Natural killer T cells, Neutrophils, Plasmacytoid dendritic cells, Type 17 T helper cells, and Type 2 T helper cells.

Next, a correlation heatmap was created to depict the correlation of infiltration abundance among these nine immune cells (Figure 9B). The results reveal that Natural killer T cells and Type 17 T helper cells show the strongest negative correlation (r value = −0.97), whereas Plasmacytoid dendritic cells and Neutrophils display the strongest positive correlation (r value = 0.96).

A correlation bubble plot was ultimately employed to illustrate the association between ECMRDEGs and the abundance of immune cell infiltration in Dataset-NP (Figure 9C). The plot demonstrated that CTSB has a notably negative correlation with Type 17 T helper cells (r = −0.97, p < 0.05), whereas TGM2 shows a significantly positive correlation with Natural killer T cells (r = 0.99, p < 0.05). Additionally, the ssGSEA algorithm was utilized with Dataset-PH to estimate the infiltration abundance of 28 different immune cell types. Following the immune infiltration analysis, a T-test was conducted for differential validation, and a comparative plot was generated to display differences in immune cell infiltration expression among various groups (Figure 9D). The findings indicated that only the CD56bright natural killer cell exhibited significant statistical differences (p < 0.05) across groups in Dataset-PH. Lastly, the relationship between Hub Genes and immune cell infiltration abundance in Dataset-PH was examined.

### hAEC-EVs accelerate the proliferation and migration of HCSCs and HCECs

**FIGURE 10:**
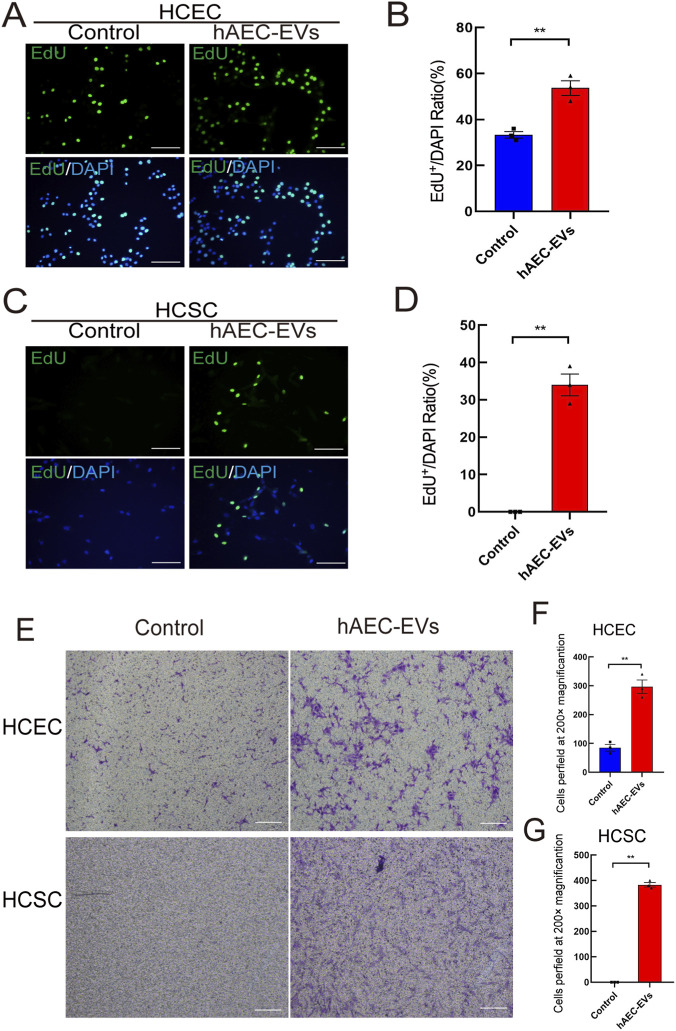
The facilitatory effect of hAEC-EVs on the proliferation and migration of HCECs and HCSCs. **(A,C)** Fluorescence images of EdU-positive staining of HCECs or HCSCs treated with PBS or hAEC-EVs (100 μg/mL). Scale bars: 100 µm. **(B,D)** Histogram representing the ratio of EdU-positive stained cells. Data are presented as the means ± SEM (n = 3). **: p < 0.01. **(E–G)** Representative images of migrated cells with crystal violet staining in the Transwell assay for 24 h. Histogram representation of the number of migrated cells in the Transwell assay. Data are presented as the means ± SEM (n = 3). **: p < 0.01.

To elucidate the cellular effects and underlying mechanisms of hAEC-EVs on HCECs and HCSCs, we investigated their influence on cell proliferation and migratory capacity *in vitro*. Cell proliferation was assessed using an EdU incorporation assay following treatment with hAEC-EVs at a concentration of 100 μg/mL. The results shown in Figures Figure10A,B–D demonstrate that exposure to hAEC-EVs markedly enhanced the proliferation of both HCECs (p < 0.01) and HCSCs (p < 0.01). In addition, hAEC-EVs significantly facilitated cell migration. Transwell assays indicated that hAEC-EVs increased the migratory activity of HCECs and stimulated HCSCs to traverse the porous membrane, as illustrated in Figures 10E–G.

## Discussion

Corneal alkali burns are characterized by high incidence and severe visual morbidity, yet effective clinical treatments remain limited. In our previous study, we demonstrated that hAEC-EVs significantly attenuate inflammation, promote re-epithelialization, improve stromal organization, and reduce scar formation in a rabbit model of corneal alkali injury (Hu et al., 2022). Building upon these findings, the present study represents a secondary, mechanism-focused investigation aimed at elucidating the ECM–related molecular programs associated with hAEC-EV–mediated corneal repair.

Proteomic and bioinformatic analyses identified ten ECM-related differentially expressed genes associated with corneal injury and hAEC-EV treatment. Among these, A2M, LAMA1, and VIT were markedly upregulated.Alpha-2-macroglobulin (A2M) helps maintain tissue structure and function post-injury by inhibiting the activity of matrix metalloproteinases (MMPs) to prevent excessive ECM degradation (OʼShea and Smith, 2014). Laminin-1 (LAMA1), a key basement membrane component, plays a central role in cell–ECM adhesion and stromal organization, and has been implicated in tissue regeneration across multiple organ systems (Nam et al., 2017; Nam et al., 2019). The upregulation of these molecules suggests that hAEC-EVs contribute to stabilizing the corneal ECM and preserving stromal architecture after injury. Differential expression analysis also identified genes downregulated by hAEC-EVs treatment, such as CTSB (Hook et al., 2022), associated with inflammation and tissue injury response. By reducing the expression of such genes, hAEC-EVs may further alleviate inflammatory responses following corneal injury, creating a more favorable microenvironment for tissue repair.

Gene set enrichment analysis (GSEA) results indicated that hAEC-EVs may support injury repair by modulating cell-ECM interactions, impacting ECM-receptor interaction and other relevant signaling pathways. This includes enhancing cellular adhesion, migration, proliferation, and differentiation—activities critical for restoring tissue functionality and transparency in the context of corneal injury. To provide direct functional evidence supporting these bioinformatic predictions, we further performed EdU proliferation and Transwell migration assays, which demonstrated that hAEC-EVs significantly promote the proliferation and migration of HCECs and HCSCs *in vitro*. These findings reinforce the GSEA-based indications that hAEC-EVs facilitate corneal repair through modulation of cellular behaviors essential for tissue regeneration.

Protein–protein interaction network analysis identified A2M, CTSB, ITGB6, LAMA1, TIMP1, and VCAN as hub genes within the ECM regulatory network. These molecules are known to participate in matrix stabilization, proteolytic control, and cell–matrix signaling, highlighting ECM remodeling as a central molecular axis underlying hAEC-EV–associated corneal repair. Immune infiltration analysis revealed that hAEC-EVs, while modulating the ECM, may also influence the tissue repair process by altering the immune microenvironment. By regulating the infiltration and activity of immune cells, hAEC-EVs can precisely modulate the inflammatory response at the injury site, creating a more ideal environment for ECM reconstruction. However, these immune infiltration findings are based on computational predictions and will require further experimental validation using approaches such as immunohistochemistry or flow cytometry in future studies. In addition, it should be noted that the bioinformatic analyses were primarily based on human gene annotations and immune signatures, and reconstruction of the entire workflow using rabbit-specific databases was beyond the scope of the present study.

Protein-level validation using Western blotting confirmed the upregulation of A2M, LAMA1, and VIT and the downregulation of CTSB, demonstrating strong concordance between proteomic screening and targeted experimental verification. Western blotting was selected for protein-level validation due to its quantitative reliability and suitability for detecting multiple ECM-related proteins in limited corneal tissue samples. Together with qPCR confirmation, these data provide robust molecular evidence supporting the regulatory effects of hAEC-EVs on ECM remodeling and strengthen the mechanistic framework proposed in this study.

Several limitations of this study should be acknowledged. Although key ECM-related genes regulated by hAEC-EVs were identified and validated at both the mRNA and protein levels, direct causal evidence linking individual ECM components to the corneal repair phenotype is lacking. Targeted mechanistic approaches, such as gene manipulation or pathway inhibition, were not performed. Thus, while our findings support an association between hAEC-EV–induced ECM remodeling and enhanced corneal repair, the precise causal roles of specific ECM molecules require further investigation in future studies. In addition, due to the limited sample size of the proteomic datasets, differential expression analyses were performed using a relatively permissive fold-change threshold, and re-analysis with more stringent cutoffs or larger cohorts was beyond the scope of the present study.

In this context, despite the comprehensive analysis of ECM-related differentially expressed genes in this study, it should be noted that certain key stromal proteoglycans essential for corneal structure and transparency, such as decorin, were not included in the downstream analyses. In the present work, candidate molecules for functional validation were selected based on an unbiased proteomic analysis with predefined statistical thresholds, and decorin did not meet the criteria for differential expression in the analyzed corneal tissues. This absence does not exclude its potential involvement in corneal repair, as decorin function may be regulated through post-translational modifications, spatial redistribution within the stroma, or time-dependent expression changes not captured under the current experimental conditions. Future studies employing targeted proteomic approaches, immunohistochemical analyses, or time-course investigations will be necessary to comprehensively elucidate the contribution of decorin and other proteoglycans to hAEC-EV–mediated corneal ECM remodeling.

## Conclusion

In conclusion, this study provides a comprehensive and experimentally validated mechanistic analysis of hAEC-EV–mediated corneal injury repair. By integrating proteomic profiling, bioinformatic analyses, protein-level validation, and functional *in vitro* assays, we demonstrate that hAEC-EVs promote corneal healing through coordinated regulation of ECM remodeling, suppression of injury-associated proteolysis, enhancement of cell proliferation and migration, and modulation of the immune microenvironment. These findings strengthen the biological rationale for hAEC-EV–based therapies and support their potential clinical application in the treatment of corneal injuries. Future studies focusing on additional ECM components and translational validation will further advance the therapeutic development of hAEC-EVs.

## Data Availability

The mass spectrometry proteomics data have been deposited to the ProteomeXchange Consortium (https://proteomecentral.proteomexchange.org) via the iProX partner repository with the dataset identifier PXD075526. All other data are included within the article.
